# Micro- and Nanoscale Flow Mechanisms in Shale Oil: A Fluid–Solid Coupling Model Integrating Adsorption, Slip, and Stress Sensitivity

**DOI:** 10.3390/nano16020144

**Published:** 2026-01-21

**Authors:** Zupeng Liu, Zhibin Yi, Guanglong Sheng, Guang Lu, Xiangdong Xing, Xinlong Zhang

**Affiliations:** 1 State Key Laboratory of Shale Oil and Gas Enrichment Mechanisms and Effective Development, Beijing 100083, China; 2 School of Petroleum Engineering, China University of Petroleum (East China), Qingdao 266580, China; 3 Exploration and Development Research Institute, Shengli Oilfield Company, SINOPEC, Dongying 257015, China; 4 College of Petroleum Engineering, Yangtze University, Wuhan 430100, China; 5 Shenzhen Branch of CNOOC (China) Co., Ltd., Shenzhen 518040, China

**Keywords:** shale oil, microscale flow mechanisms, fluid–solid coupling, adsorption effect, slip effect, stress sensitivity

## Abstract

Shale oil reservoirs are complex multi-scale nanoporous media where fluid transport is governed by coupled micro-mechanisms, demanding a robust modeling framework. This study presents a novel fluid–solid coupling (FSC) numerical model that rigorously integrates the three primary scale-dependent transport phenomena: adsorption in organic nanopores, slip effects in inorganic micropores, and stress-sensitive conductivity in fractures. The model provides essential quantitative insights into the dynamic interaction between fluid withdrawal and reservoir deformation. Simulation results reveal that microstructural properties dictate the reservoir’s mechanical stability. Specifically, larger pore diameters and higher porosity enhance stress dissipation, promoting long-term stress relaxation and mitigating permeability decay. Crucially, tortuosity governs the mechanical response by controlling pressure transmission pathways: low tortuosity causes localized stress concentration, leading to rapid micro-channel closure, while high tortuosity ensures stress homogenization, preserving long-term permeability. Furthermore, high fracture conductivity induces a severe, heterogeneous stress field near the wellbore, which dictates early-stage mechanical failure. This work provides a powerful, mechanism-based tool for optimizing micro-structure and production strategies in unconventional resources.

## 1. Introduction

Shale oil, as a crucial unconventional resource during the global energy transition, holds significant strategic importance for ensuring energy security [1]. However, shale oil reservoirs are fundamentally distinct from conventional reservoirs; they are complex “organic-inorganic composite nanoporous media” [2]. Characterized by low porosity, low permeability, strong heterogeneity, and high stress sensitivity, the development of these reservoirs involves a continuous coupling between fluid flow and reservoir deformation [3,4,5]. Consequently, traditional seepage theory often fails to accurately describe the complex dynamic responses within these systems. To achieve efficient development, it is imperative to understand the complex flow mechanisms at the micro- and nanoscale.

The microscopic pore system of shale oil reservoirs constitutes a multi-scale, multi-physics coupled entity composed of organic pores, inorganic pores, and microfractures [6]. Distinct transport mechanisms govern fluid behavior within these diverse storage spaces. Organic nanopores predominantly develop within kerogen with dimensions ranging from nanometers to tens of nanometers [7,8]. These pores serve not only as the primary storage space but also as a domain governed by “surface phenomena” such as van der Waals forces [9]. Fluid transport here is dominated by adsorption and desorption mechanisms, rather than simple Darcy flow [10,11]. While inorganic pores range up to several hundred nanometers, these pores act as flow channels for free-phase oil [7,8]. However, due to fluid–solid interactions, the boundary layer effect and fluid slip at the pore walls become non-negligible factors influencing microscale flow, particularly in hydrophilic mineral pores [12,13]. And for microfractures acting as preferential seepage pathways, their conductivity far exceeds that of the pore matrix [14]. Crucially, their aperture width and conductivity are highly sensitive to the effective stress field, exhibiting strong stress-dependent permeability [15].

The challenge lies in the fact that these distinct microscale physical effects (adsorption in nanopores and slip in micropores) are strongly coupled with the macroscopic geomechanical stress field. As demonstrated by Wang et al. [16], the coupling of nanoscale effects (e.g., wall slippage) with stress sensitivity renders fluid transport mechanisms considerably more complex than in conventional reservoirs.

Numerous scholars have conducted in-depth research to address these complexities. Lei et al. [17] elucidated fluid flow patterns in cores of varying petrophysical properties using CT scanning and NMR technology. Sang [18] systematically analyzed the reservoir characteristics and mobility conditions for shale oil. Focusing on microscopic forces, Liu [9] constructed a nanoporous flow model incorporating van der Waals forces, revealing their impact on seepage and residual oil. Wang [8] employed molecular dynamics simulations to uncover the effects of pore-throat size and mineral composition on occurrence states. Furthermore, Li et al. [19] confirmed through low-rate seepage experiments that boundary layer effects and slip length are key factors controlling low-velocity non-Darcy flow, while Zhao et al. [20] established a nanoscale transport model accounting for wettability and stress sensitivity.

These studies have laid a solid foundation for understanding shale oil microscale flow. However, most existing research tends to focus on singular microscale phenomena—either emphasizing adsorption effects or stress sensitivity in isolation—and lacks a unified model that can simultaneously integrate and dynamically couple the “adsorption-dominated flow” in organic nanopores, the “slip-dominated flow” in inorganic pores, and the “stress-sensitive conduction” in fracture networks. Consequently, it remains difficult to accurately predict the dynamic evolution of these competing physical mechanisms and their combined impact on reservoir performance throughout the development lifecycle.

To address these challenges, this study establishes a novel microscale fluid–solid coupling numerical model that integrates multi-scale pore structure characterization, partitioned flow modeling, and dynamic coupling techniques. This work aims to achieve three main objectives. The first is to strictly distinguish and quantify the differential impacts of adsorption effects in organic pores, slip effects in inorganic pores, and stress-sensitive conductivity in fractures on overall flow behavior. The goal is to establish a dynamic relationship linking pore pressure, effective stress, and permeability evolution, thereby revealing the underlying microscopic mechanisms of fluid–solid interaction. The last is to build a “Seepage Field–Deformation Field–Parameter Update” closed-loop coupled model, enabling accurate predictive simulation of dynamic reservoir behavior and providing theoretical guidance for optimizing shale oil development.

## 2. Materials and Methods

### 2.1. Multi-Scale Pore Structure Characterization and Physical Model

**Figure 1:**
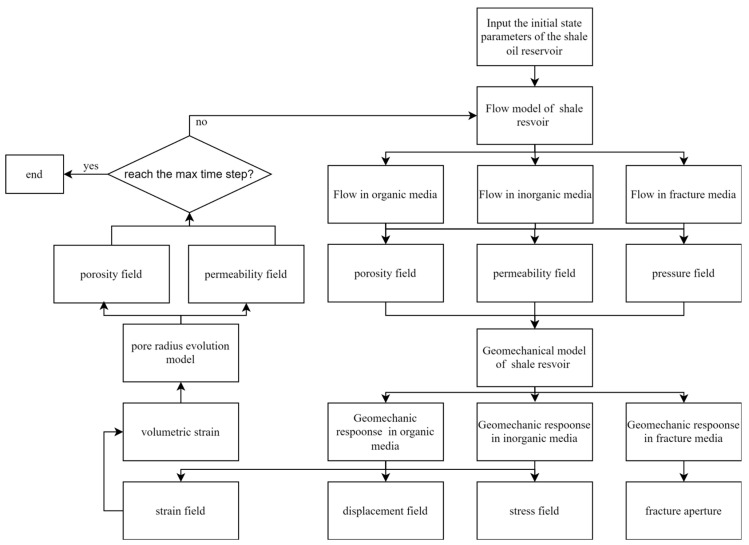
Microscale fluid–structure interaction process in shale reservoirs.

Shale oil reservoirs are quintessential micro- to nano-scale porous media characterized by extreme heterogeneity [21]. Unlike conventional reservoirs, the storage space in shale is a composite system comprising three distinct media types: organic nanopores, inorganic micropores, and macroscopic microfractures (Figure 1). Accurately distinguishing the distinct fluid storage and transport mechanisms within these media is the foundation of this study.

Organic pores predominantly develop within kerogen; these pores typically range from nanometers to tens of nanometers [7,8]. Due to the strong affinity between organic matter and hydrocarbons, fluid behavior here is governed by surface thermodynamics. The pore surfaces act as the primary sites for adsorption via van der Waals forces, forming a dense adsorbed phase that significantly alters the effective pore diameter [7,8]. Inorganic pores (Microscale Pathways), located between mineral grains (e.g., quartz and clay), are generally larger (up to hundreds of nanometers). While they provide channels for free-phase oil, the fluid–solid interaction at the inorganic walls—specifically the boundary layer effect—introduces non-Darcy flow characteristics such as slippage, distinguishing them from simple pipe flow [22]. Whether natural or hydraulically induced, fractures form a high-permeability network that traverses both organic and inorganic matrices [15]. While their flow follows Darcy’s law, their aperture widths are highly sensitive to effective stress variations, making them the primary locus for stress-dependent permeability evolution during depletion [23,24].

To accurately represent the non-Newtonian flow behavior of shale oil in confined pores, the constant viscosity assumption is replaced with a constitutive model. The Herschel–Bulkley model is adopted for its capability to describe two critical rheological phenomena pertinent to shale oil: yield stress and shear-thinning behavior. It is characterized by a flow behavior index *n* less than 1.

Consequently, conventional single-porosity Darcy models fail to capture this complexity. This study establishes a partitioned fluid–solid coupling (FSC) model that mathematically isolates and integrates the distinct transport mechanisms—adsorption in organic matter, slip in inorganic matrix, and stress sensitivity in fractures.

### 2.2. Mathematical Modeling of Microscale Shale Oil Flow

#### 2.2.1. Slip-Dominated Flow in Inorganic Pores

Inorganic pores in shale are predominantly hydrophilic. In these confined micro-channels, the interaction between fluid molecules and the pore wall creates a distinct velocity profile. While water molecules may form a no-slip boundary layer due to hydrogen bonding [25], shale oil exhibits a measurable slip phenomenon at the wall, reducing flow resistance.

To capture this, we abandon the classical no-slip assumption and model the flow using a modified Hagen–Poiseuille equation incorporating slip length. Critically, to account for the non-Newtonian rheology of shale oil, we introduce the Herschel–Bulkley constitutive model. The shear stress (*τ*) is related to the shear rate (*γ*^n^) by

(1) $$\tau =\tau_{0}+K\cdot \gamma^{n}$$

where *τ*_0_ is the yield stress (Pa), *K* is the consistency coefficient (Pa·s^n^), and *n* is the flow behavior index (dimensionless). For shear-thinning shale oils, *n* < 1. The apparent viscosity, which replaces the constant bulk viscosity, is then defined as:

(2) $$\mu_{b,\mathrm{app}}=\tau /\gamma =\tau_{0}/\gamma +K\cdot \gamma^{n-1}$$

where *v_b_* denotes the flow velocity of bulk-phase shale oil (m/s); *μ*_b,app_ represents the shear-dependent apparent viscosity (mPa·s), calculated from the Herschel–Bulkley model; α is the ratio of adsorbed-layer fluid viscosity to boundary-layer fluid viscosity (dimensionless); *R* signifies the radius of the inorganic pore (m); and *λ* designates the slip length (m).

The flow velocity is enhanced by the adsorbed-to-boundary layer viscosity ratio and the slip effect, while also being modulated by the shear-dependent apparent viscosity. This relationship is derived from the momentum conservation for steady, laminar flow of a non-Newtonian fluid in a cylindrical capillary, using a two-layer fluid model with a Navier slip boundary condition. Based on this, we obtain the following expression:

(3) $$\left\{\begin{array}{ll} v_{b}(r)=\left[\frac{R^{2}-r^{2}}{4\beta_{\mathrm{in}}}+\frac{\alpha -1}{4\alpha }{\left(R-\delta \right)}^{2}+\frac{\lambda R}{2\beta_{\mathrm{in}}}\right]\frac{\Delta p}{\mu_{b,\;\mathrm{app}}L}, & 0\leq r\leq R-\delta \\ v_{\delta }(r)=\left(\frac{R^{2}-r^{2}}{4\beta_{\mathrm{in}}}+\frac{\lambda R}{2\beta_{\mathrm{in}}}\right)\frac{\Delta p}{\mu_{b,\;\mathrm{app}}L}, & R-\delta \leq r\leq R \end{array}\right.$$

where *v_b_* denotes the flow velocity of bulk-phase shale oil (m/s); *α*_in_ is the ratio of adsorbed-layer fluid viscosity to boundary-layer fluid viscosity (dimensionless); *R* signifies the radius of the inorganic pore (m); *λ* designates the slip length (m); Δ*p* indicates the pressure difference across the pore (Pa); *L* is to the pore length (m); and *δ* is the thickness of the adsorption layer. Notably, based on molecular dynamics simulations revealing that wall effects typically extend over approximately two molecular layers of hydrocarbon fluids, a constant boundary layer thickness of δ = 1.0 nm is adopted in this study [20,25]. This value provides a characteristic thickness to quantify how property heterogeneity influences slip-enhanced flow.

Critically, the slip length is not a fixed constant but is intrinsically dependent on the physicochemical properties of the pore surface. To capture this essential dynamic, *λ* is calculated based on the specific wettability of the pore wall. For the inorganic (typically hydrophilic) and organic (typically hydrophobic) pores, we employ an empirical correlation derived from experimental and molecular dynamics simulation data [26], which expresses slip length as a function of the contact angle:

(4) $$\lambda =\frac{C}{{\left(1+\cos\theta \right)}^{2}}$$

where *C* is a fitting coefficient consolidated from prior studies, and *θ* is the contact angle that quantitatively represents the wettability of the fluid–solid system. This formulation ensures that the slip effect is dynamically coupled to the pore type: a larger contact angle (stronger hydrophobicity/oil-wetness) yields a larger *λ*, significantly enhancing flow, whereas a smaller contact angle (hydrophilicity) results in a diminished slip length. This approach provides a more physically grounded representation of interfacial transport than using a single averaged value.

The volumetric flow rate for each phase is obtained by integrating the velocity profile over its respective cross-sectional area:

(5) $$Q=\int_{0}^{R}2\pi rv(r)dr=\int_{0}^{R-\delta }2\pi rv_{b}(r)dr+\int_{R-\delta }^{R}2\pi rv_{s}(r)dr$$

This formulation explicitly accounts for the boundary layer effect, ensuring that the transition from nanoscale confinement to microscale bulk flow is physically consistent. By integrating this over the effective porosity and tortuosity of the inorganic matrix, we derive the macroscopic volumetric flow rate for the inorganic continuum:

(6) $$\left\{\begin{array}{l} Q_{b}=\frac{\pi \rho_{b}\Delta p}{8\beta \mu_{b,\mathrm{app}}L}{\left(R-\delta \right)}^{2}\left[(4R\delta -2\delta^{2})+\beta_{\mathrm{in}}{\left(R-\delta \right)}^{2}+4\lambda R\right]\\ Q_{\delta }=\frac{\pi \rho_{\delta }\Delta p}{8\beta \mu_{b,\mathrm{app}}L}(2R\delta -\delta^{2})(2R\delta -\delta^{2}+4\lambda R) \end{array}\right.$$

The total apparent mass flow rate at the representative elementary volume (REV) scale is calculated by summing the contributions from all inorganic pores, accounting for the rock’s porosity (*ϕ*) and the tortuosity (*τ*) of the flow paths:

(7) $$q_{m}=\frac{\phi }{\tau }(\rho_{b}Q_{b}+\rho_{s}Q_{s})=\frac{\pi \phi \Delta p}{8\beta_{\mathrm{in}}\tau \mu_{b}L}\left\{\begin{array}{c} \rho_{b}{\left(R-\delta \right)}^{2}\left[4R\delta -2\delta^{2}+\alpha_{\mathrm{in}}{\left(R-\delta \right)}^{2}+4\lambda R\right]\\ +\rho_{w}(2R\delta -\delta^{2})\left(2R\delta -\delta^{2}+4\lambda R\right) \end{array}\right\}$$

#### 2.2.2. Adsorption-Dominated Transport in Organic Pores

Flow within organic nanopores is fundamentally different. The nanoconfinement effect, resulting from the high surface-to-volume ratio in these ultra-narrow pores, renders physical adsorption a dominant resistance mechanism. Strong dipole interactions and van der Waals forces cause hydrocarbon molecules to adhere to the kerogen walls, forming a quasi-stationary adsorbed layer that effectively reduces the channel radius available for flow [27].

In organic nanopores, the adsorbed phase is modeled using a two-layer approach to account for rheological complexity. The effective pore radius *R*_e_ is defined as:

(8) $$R_{e}\;=\;R-h$$

where *R* is the true pore radius (m), and *h* is the thickness of the immobile adsorbed layer (m). The total adsorbed layer thickness *δ*_ads_ is given by

(9) $$\delta_{\mathrm{ads}}=h+\delta_{v}$$

where *δ*_v_ is the thickness of the viscous interfacial layer (m). The immobile layer thickness *h* is typically in the range of 0.5–2 nm for organic-rich shale nanopores, based on molecular dynamics simulations [28]. The viscous interfacial layer thickness *δ*_v_ is calculated from the Langmuir adsorption isotherm combined with viscosity correlations:

(10) $$\delta_{v}=\delta_{\max}\frac{p/p_{L}}{1+p/p_{L}}-h$$

where *δ*_max_ is the maximum thickness of the viscous interfacial layer (m), *p*_L_ is the Langmuir pressure (Pa), and *p* is the pore pressure (Pa). The flow of the free phase within the reduced effective radius *R*_e_ is then governed by a modified Hagen–Poiseuille equation accounting for the high viscosity of the interfacial layer, characterized by the viscosity ratio $\alpha_{\mathrm{or}}=\mu_{\mathrm{ads}}/\mu_{b}$.

Since the adsorbed phase is immobile (no-slip), the transport equation for organic pores is strictly governed by the flow of the free phase within this reduced effective radius:

(11) $$q_{or}=\frac{\pi \phi \Delta p}{8\tau \alpha_{\mathrm{or}}\mu_{b}L}\left\{\begin{array}{l} {\left(R_{e}-\delta_{v}\right)}^{2}\left[4R_{e}\delta_{v}-2{\delta_{v}}^{2}+\alpha_{\mathrm{or}}{\left(R_{e}-\delta_{v}\right)}^{2}\right]\\ +{\left(2R_{e}\delta_{v}-{\delta_{v}}^{2}\right)}^{2} \end{array}\right\}$$

#### 2.2.3. Stress-Dependent Seepage in Fractures

Unlike the matrix pores, flow in microfractures is pressure-driven and follows Darcy’s law, as the aperture size renders molecular wall effects negligible [29]:

(12) $$q_{f}=-\frac{k_{f}A}{\mu }\frac{dp}{dx}$$

However, the critical micro-mechanism here is geomechanical closure. As fluid is extracted, the decline in pore pressure increases the effective stress on the fracture faces, causing them to close. This nonlinear reduction in conductivity is the primary driver of production decline in the early stages and is coupled via a fracture compressibility term in the geomechanical model.

### 2.3. Geomechanical Model for Reservoir Deformation

To close the loop between fluid withdrawal and rock deformation, we employ Biot’s poroelastic theory. The shale is treated as a dual-continuum (organic/inorganic) with distinct mechanical properties. High organic content implies lower stiffness and greater deformation susceptibility, while inorganic minerals provide structural rigidity.

The constitutive relations couple the strain tensor, effective stress, and pore pressure:

(13) σ=C:ε−αpI

(14) ∇⋅σ+f=0

where ***σ*** is the Cauchy stress tensor (Pa), **C** is the fourth-order elastic stiffness tensor (Pa), *ε* is the infinitesimal strain tensor (dimensionless), *α*_B_ is the Biot coefficient (dimensionless), *p* is the pore fluid pressure (Pa), **I** is the second-order identity tensor, and **f** is the body force vector (Pa/m).

The heterogeneity of organic partitioning and inorganic partitioning was considered through equilibrium dynamics. The momentum balance equation under quasi-static conditions is

(15) $$\nabla \cdot (C_{in}:\varepsilon_{in}+C_{or}:\varepsilon_{in})-\alpha \nabla p+f=0$$

The global deformation is solved by balancing momentum, explicitly accounting for the heterogeneity between organic and inorganic partitions.

For the fracture network, deformation is modeled as a direct function of normal effective stress, governed by the fracture compressibility coefficient:

(16) $$\frac{w_{f}}{w_{f0}}=1-c_{f}(\sigma_{n}-\sigma_{n0})$$

where *w_f_* and *w_f_*_0_ are the current and initial hydraulic fracture aperture (m), respectively; *c_f_* is the fracture compressibility coefficient (Pa^−1^); and *σ*_n_ and *σ*_n0_ are the current and initial effective normal stress acting on the fracture plane (Pa).

### 2.4. Dynamic Fluid–Solid Coupling Strategy

When subjected to external loading, shale reservoirs undergo microstructural geo-metric alterations. To solve the FSC mathematical model, it is essential to integrate cou-pled seepage-stress equations alongside the governing equations for rock deformation and fluid flow. These equations explicitly quantify the dynamic interdependencies between key seepage parameters and geomechanical parameters within shale reservoirs. This study implements a bidirectional dynamic feedback mechanism for coupling the seepage and stress fields. For the shale matrix (organic/inorganic pores), pore pressure and permeability fields at the current timestep are solved using the shale oil flow equations specific to organic/inorganic pores. Matrix porosity is dynamically updated based on the volumetric strain (ε_v_) of the shale matrix. For the fracture system, fracture conductivity is dynamically adjusted according to effective stress variations, characterized by the fracture compressibility coefficient. Following Huang et al. [15], the dynamic evolution of shale porosity during coupled seepage–stress processes can be characterized by

(17) $$\phi_{m}=\frac{\phi_{m0}+\varepsilon_{V}}{1+\varepsilon_{V}}$$

(18) $$\varepsilon_{V}=\varepsilon_{\mathrm{xx}}+\varepsilon_{\mathrm{yy}}+\varepsilon_{\mathrm{zz}}$$

where *ϕ*_m_ denotes the matrix porosity (dimensionless); *ϕ*_m0_ denotes the initial matrix porosity (dimensionless); and *ε*_v_ denotes the volumetric strain, reflecting the rate of change in rock geometry (dimensionless).

The permeability evolution of the shale matrix can be derived using the Kozeny–Carman equation, with post-deformation permeability expressed as:

(19) $$k_{app}=\frac{k_{m0}}{\left(1+\varepsilon_{V})(\tau_{0}/\gamma +K\cdot \gamma^{n-1}\right)}{\left(1+\frac{\varepsilon }{\phi_{m0}}\right)}^{3}$$

Pore diameters in both organic and inorganic domains are also affected by reservoir deformation. Studies indicate a dynamic correlation among matrix porosity, permeability, and pore diameter [30]. This work employs the following equation to quantify shale pore diameter evolution:

(20) $$R^{2}=c_{p}\frac{k_{m}}{\phi_{m}^{b_{p}}}$$

where *R* denotes the pore radius (m) and *c_p_* and *b_p_* denotes empirical stress sensitivity coefficients associated with the shale matrix (dimensionless).

The MINC (Multiple Interacting Continua) model provides a methodology for calculating permeability in microfractures, where fracture permeability is primarily governed by fracture aperture (*w*). According to the MINC model, fracture permeability evolves with aperture as:

(21) $$k_{f}=k_{f0}{\left(\frac{w_{f}}{w_{f0}}\right)}^{3}$$

Coupling between the seepage field and stress field is achieved through a bidirectional dynamic feedback loop. The computational workflow comprises three sequential phases, as illustrated in Figure 1. Microscale fluid–structure interaction process in shale reservoirs:

(1) Seepage-Dominated Phase: Solve the flow equations to obtain the pore pressure distribution and permeability field (*k*) for the current timestep. Output these results to the geomechanical model.
(2) Geomechanical Response Phase: Compute the displacement field, stress field, and volumetric strain (*ε*_v_) of the shale reservoir using the stress equilibrium equations. Subsequently, update key parameters—including organic/inorganic pore diameters, matrix permeability, and fracture aperture—based on the reservoir’s deformation characteristics.
(3) Parameter Update and Feedback Phase: Reconstruct the permeability field using the updated porosity and fracture aperture through pore evolution models. Feed this updated permeability field back into the seepage equations for the next timestep.

## 3. Model Validation

**Figure 2:**
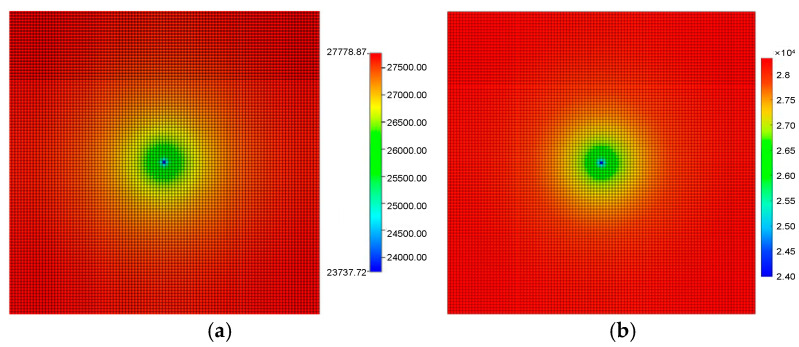
Pressure distribution after 1440 days of depletion development: (**a**) is the simulation result of the CMG; (**b**) is the simulation result of the model proposed in this paper.

To ensure the proposed microscale FSC model accurately captures the dynamic interplay between the seepage field and the reservoir deformation field, we performed a benchmark validation against the industry-standard commercial simulator, CMG (Computer Modelling Group, version 2022, Canada). The developed model was first validated against the widely used commercial simulator CMG (IMEX) under isothermal, Newtonian fluid conditions. This comparison, as shown in Figure 2, demonstrates excellent agreement in pressure and saturation fields. It is crucial to note that this step primarily verifies the correctness of the core numerical solver and the implementation of fundamental conservation laws and two-phase flow parameters (e.g., relative permeability) within our framework.

A simplified single-layer homogeneous reservoir model was established to isolate the solver’s numerical performance from the complexities of heterogeneity. The model dimensions are set to 515 m × 505 m × 10 m. The reservoir operates under depletion development with a central production well maintained at a constant bottom-hole pressure (BHP) of 20 MPa for a continuous period of 1440 days. The boundaries are initially set to zero displacement, with the bottom boundary fixed to simulate realistic confinement. The key fluid properties and geomechanical parameters used in the simulation are summarized in Table 1.

The validation focuses on two critical aspects: production performance and spatial field evolution (pressure and displacement). The simulation results demonstrate great agreement between the proposed microscale model and the CMG simulator. The cumulative production rates predicted using both methods match closely, achieving a consistency of 98%. This high degree of correlation confirms the accuracy of the mass balance equations and the flow solver within our algorithm

To verify the fluid–solid coupling algorithm, we compared the spatial distributions of reservoir states after 1440 days of production. The pore pressure distribution calculated using the proposed model (Figure 2b) is nearly identical to the CMG result (Figure 2a). Both models accurately capture the pressure funnel expanding from the central wellbore, validating the seepage solver’s performance.

**Figure 3:**
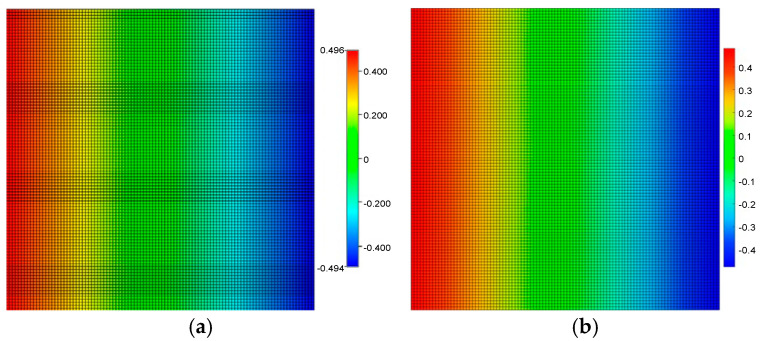
X-direction displacement distribution after 1440 days of depletion development: (**a**) is the simulation result of the CMG; (**b**) is the simulation result of the model proposed in this paper.

The X-direction displacement field, which reflects the rock matrix’s mechanical response to pressure depletion, also shows high consistency. The proposed model (Figure 3b) reproduces the deformation patterns observed in the CMG geomechanical module (Figure 3a), confirming that the coupling term correctly translates pore pressure changes into volumetric strain.

The successful validation of both production metrics and coupled physical fields (pressure and displacement) confirms the accuracy and reliability of the developed methodology. This robust numerical framework provides a solid basis for the subsequent sensitivity analysis of microscale mechanisms, such as pore diameter effects and tortuosity-induced stress variations.

**Figure 4:**
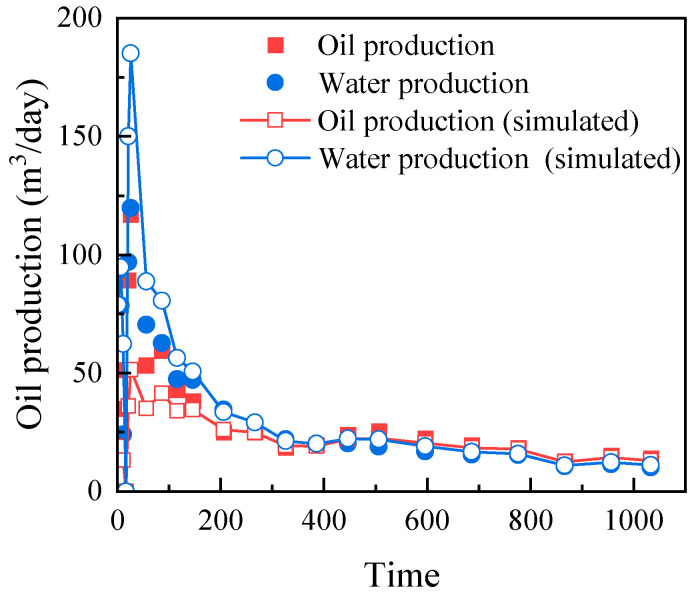
History matching results of the daily production rate of the shale oil well.

To assess the practical predictive capability of the complete model integrating slip, adsorption, and stress sensitivity, history matching was performed on a horizontal shale oil well from an oilfield in China. The well was produced under depletion for 1000 days. As shown in Figure 4, the model achieves an excellent match with the field data. The oil production rate fitting accuracy is 93.3%, and the water production rate fitting accuracy is 92.6%. This successful match indicates that the integrated model, incorporating the proposed microscale mechanisms, can reliably reproduce key field-observed production trends, providing confidence in its use for performance analysis and forecasting.

While field history matching provides strong indirect validation of the integrated model’s output, we recognize that direct, pore-scale experimental validation of the individual slip and adsorbed layer mobility mechanisms—for instance, via controlled microfluidic experiments—remains a valuable target for future research to further constrain the sub-model parameters under reservoir conditions.

## 4. Results and Discussion

The validated microscale FSC model is now applied to conduct a detailed sensitivity analysis, focusing on how key microscopic and macroscopic parameters govern the non-linear coupling mechanisms in shale oil reservoirs. This analysis provides quantitative insights into the pore-level physics controlling long-term production.

### 4.1. The Role of Microscale Pore Radius

**Figure 5:**
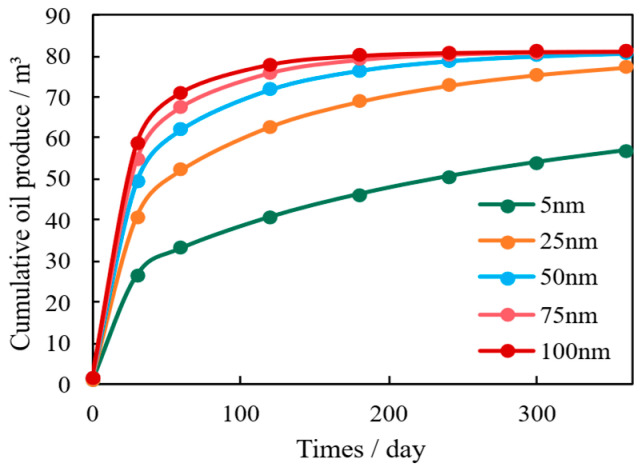
Comparison of Shale Oil Production under Different Pore Sizes.

The intrinsic pore radius is a critical microstructural parameter, dictating the relative strength of interface effects (slip and adsorption) versus bulk flow. Simulation results show that increasing the average pore diameter significantly enhances both initial production rate and cumulative oil production (Figure 5). This is primarily because larger pores reduce the surface-to-volume ratio, minimizing flow resistance caused by boundary layer effects and enhancing flow efficiency [31].

**Figure 6:**
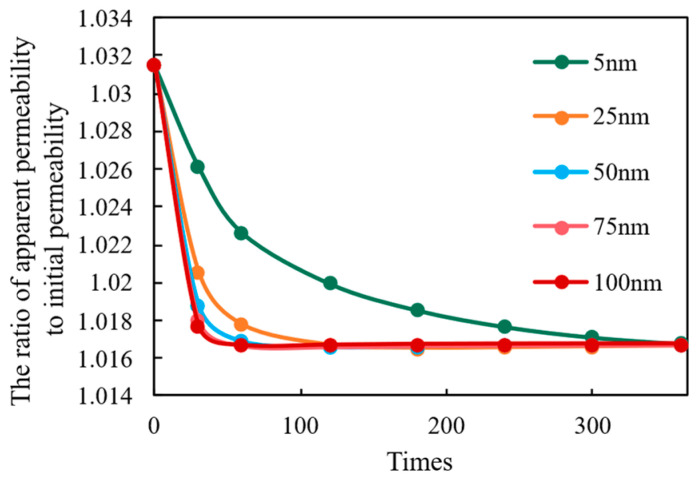
Permeability Ratio (k/k0) Evolution Under Different Pore Diameters.

However, pore diameter also influences mechanical stability. As shown in the permeability decay curves (Figure 6), while larger pores accelerate the initial pressure drop, they simultaneously facilitate more efficient pressure dissipation throughout the matrix. This rapid pressure equalization enables better long-term stress relaxation across the reservoir, which ultimately mitigates the overall degree of permeability attenuation. Conversely, reservoirs with smaller pores exhibit limited stress dissipation capacity, leading to a higher final percentage of permeability loss (approximately 1.55% for 5 nm pores versus 1.45% for 100 nm pores in this model). Thus, optimizing microscale pore diameter is essential to balance initial flow efficiency with long-term mechanical stability.

### 4.2. Fracture Conduction and Heterogeneous Micro-Stress

**Figure 7:**
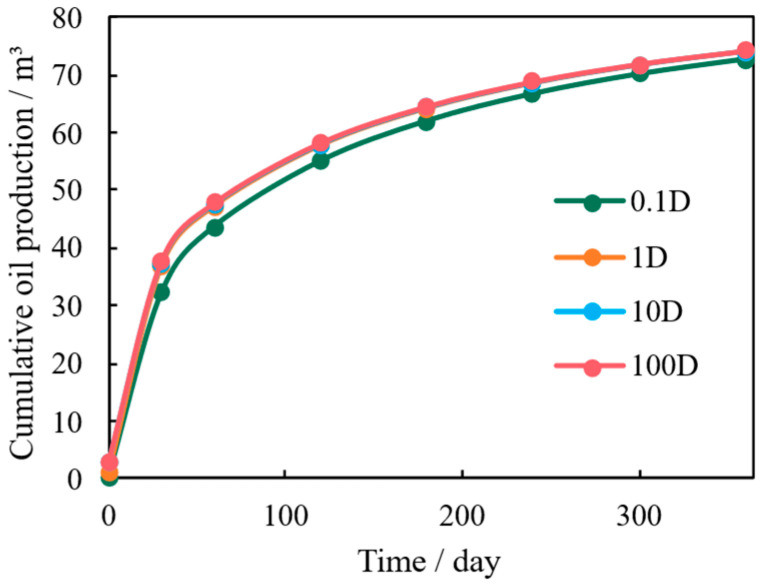
Cumulative Oil Production Under Varying Fracture Permeability.

The initial permeability of the fracture network acts as the primary macro-conduit for fluid withdrawal from the low-permeability matrix. Figure 7 shows that increasing the fracture permeability significantly boosts the initial production rate. However, the benefit to cumulative oil production exhibits a strong diminishing return. The incremental gain becomes negligible once the fracture permeability exceeds approximately 1 mD, as evidenced by the nearly identical cumulative production curves for fracture permeabilities of 1 mD and 100 mD.

This behavior stems from the coupled flow dynamics between the fracture and the matrix. In shale reservoirs, the matrix permeability is extremely low. When the fracture conductivity surpasses the matrix’s ability to supply fluid, the flow resistance within the fracture becomes negligible compared to that of the matrix. Consequently, further increasing the fracture permeability does not enhance the overall flow capacity of the system, which becomes constrained by the matrix supply. Therefore, optimizing fracture conductivity requires balancing it with the matrix deliverability, rather than pursuing maximization indefinitely.

**Figure 8:**
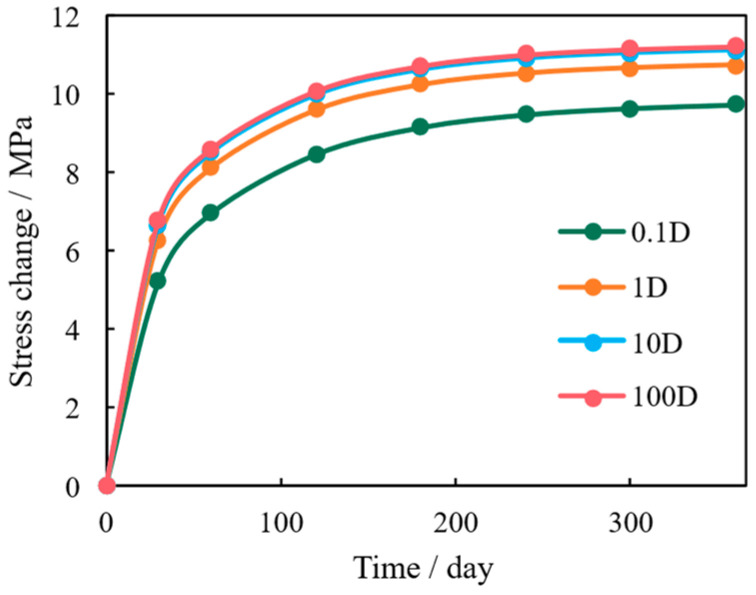
Stress Variation Under Varying Fracture Permeability.

**Figure 9:**
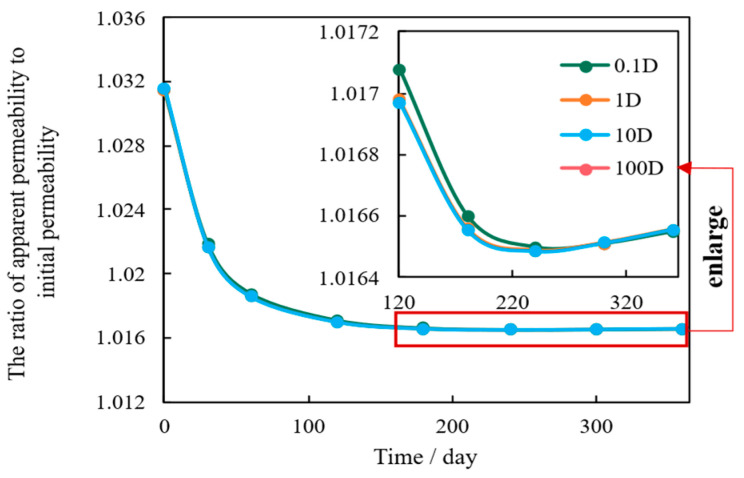
Ratio of Permeability to Initial Permeability Under Varying Fracture Permeability.

This diminishing return is rooted in the coupling mechanism. High fracture permeability creates a steeper pressure gradient near the fracture faces, inducing severe, localized stress buildup in this region (Figure 8 and Figure 9). The rapid pressure depletion translates immediately into a significant increase in effective stress, causing the fracture aperture to close quickly. This heterogeneous stress attenuation mechanism—where the micro-stress concentration near the fracture dominates—demonstrates that enhancing macro-conduction must be balanced against the induced micro-mechanical failure, leading to rapid permeability decay.

### 4.3. Porosity and Stress Dissipation Capacity

**Figure 10:**
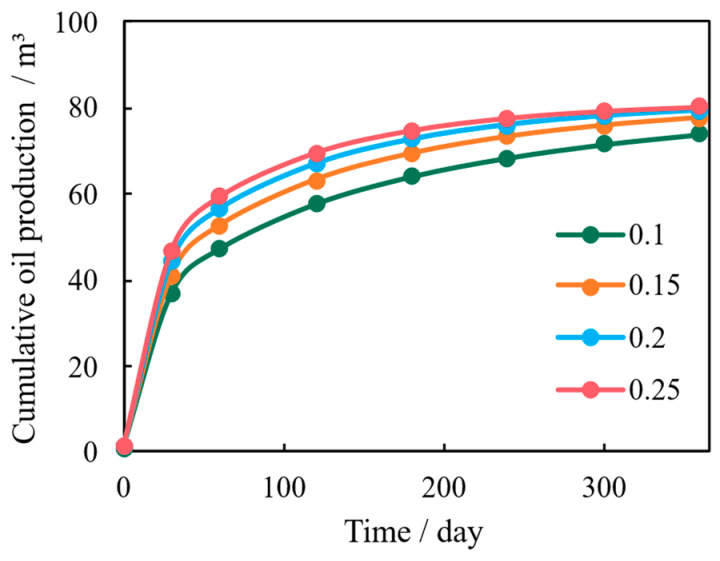
Cumulative Shale Oil Production Under Varying Porosity Conditions.

Porosity serves as both the fluid storage capacity and the reservoir’s mechanical buffering capacity. The simulation results in Figure 10 show a general positive correlation between porosity and cumulative production. Mechanistically, higher porosity initially leads to greater reservoir compressibility, which exacerbates the stress sensitivity during the early stages of production.

**Figure 11:**
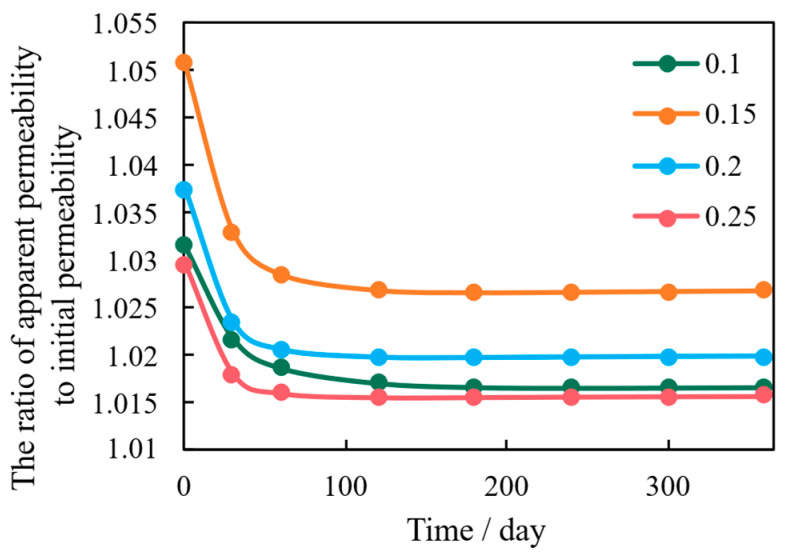
Evolution of Reservoir Matrix Permeability Under Varying Porosity Conditions.

However, in the long term, higher porosity provides a greater volume fraction for stress distribution and acts as a better pathway for fluid pressure transmission, promoting stress relaxation and dissipation [32]. This dual effect leads to the observation that while very low porosity models show severe permeability attenuation (Figure 11), there is an optimal porosity range (*ϕ* = 0.2 to 0.25) that achieves the best balance between initial deformation and long-term stress stability, resulting in maximized cumulative production.

### 4.4. Tortuosity and Stress Path Diversification

**Figure 12:**
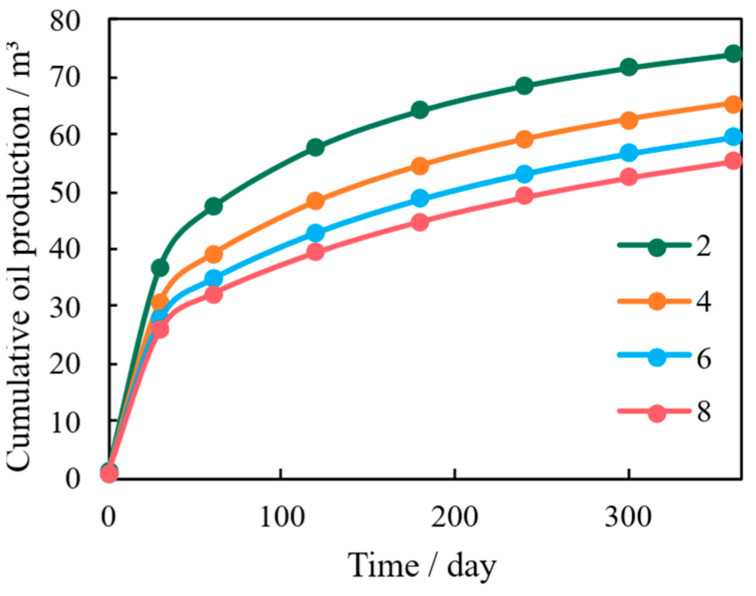
Cumulative Oil Production Under Varying Tortuosity Conditions.

The tortuosity of the porous media controls the complexity and length of the flow path, fundamentally influencing how pressure and stress are transmitted through the micro-channels. In this sensitivity analysis, the tortuosity (τ), defined as the ratio of the actual microscopic flow path length to the macroscopic sample length, is varied over a representative range for shale systems (τ = 2, 4, 6 and 8). The typical geometric tortuosity measurements 3–8 in tight shales [33,34], while the lower and upper bounds represent simplified and highly convoluted pore networks, respectively. Figure 12 shows that models with lower tortuosity exhibit higher initial production rates because the flow paths are shorter and straighter.

**Figure 13:**
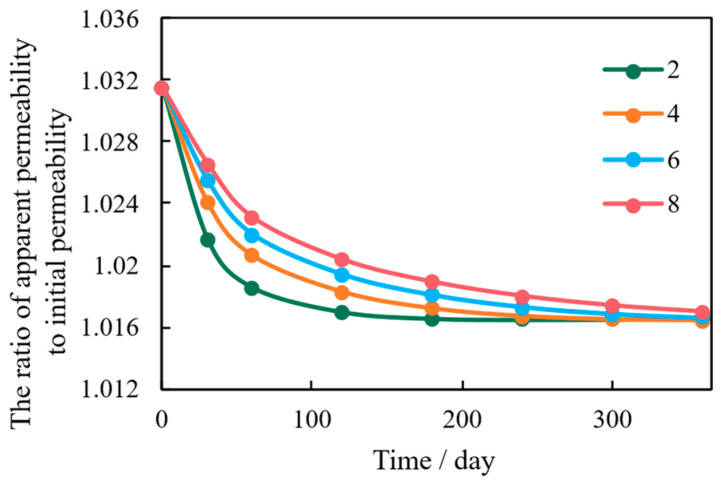
Correlation Between Apparent Permeability and Initial Permeability Under Varying Tortuosity Conditions.

However, this advantage is short-lived. Low tortuosity leads to the concentration of pressure and stress drops along these direct flow lines (Figure 13). This localized stress concentration severely limits the rock’s load-bearing capacity, resulting in rapid micro-channel closure and the highest percentage of long-term permeability decline. Conversely, high tortuosity forces fluid energy and stress to be dispersed and homogenized over a wider cross-sectional area, effectively mitigating localized mechanical failure. This stress path diversification mechanism ensures sustained permeability and contributes significantly to the reservoir’s long-term stability, despite slightly lower initial flow rates.

### 4.5. Impact of Adsorption Dynamics on Production

**Figure 14:**
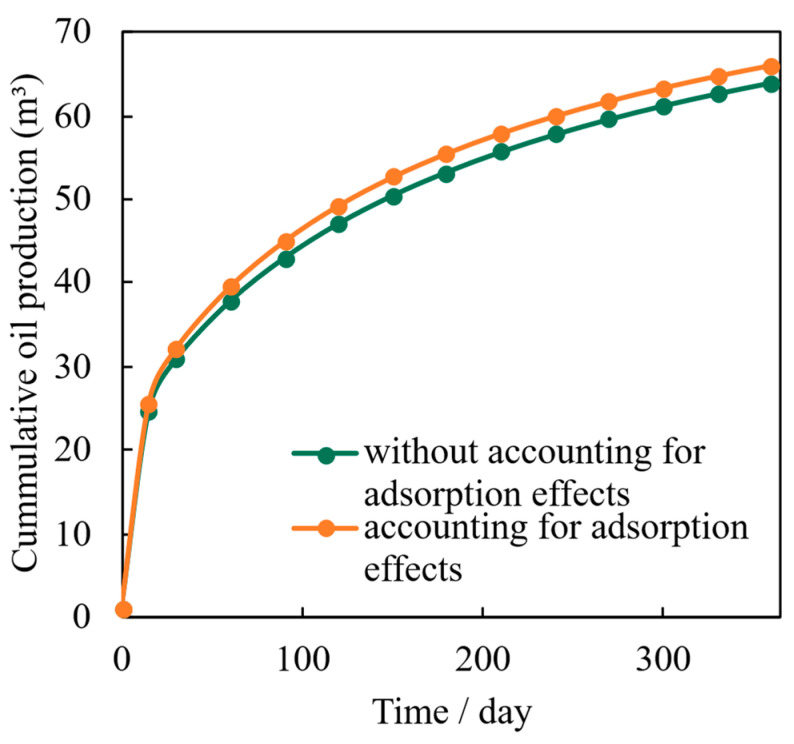
Comparison of cumulative oil production predicted using the numerical model with and without accounting for adsorption dynamics in organic nanopores.

To isolate and quantify the contribution of adsorption-related mechanisms to shale oil production, a direct comparative simulation was performed. Figure 14 presents the cumulative oil production over 360 days from two models: one incorporating the full two-layer adsorption dynamics for organic pores and one where these effects are neglected, treating organic pores similarly to inorganic pores with slip-only flow.

The results demonstrate a consistent and growing divergence between the two forecasts. While the initial production rates are similar, the model accounting for adsorption predicts higher cumulative output at every subsequent time step. By the end of the simulation period, the adsorption-aware model forecasts an ultimate recovery approximately 3.4% higher than the model that ignores adsorption.

This difference arises from the dynamic representation of the adsorbed phase. Unlike a simple, static reduction in pore radius, our model accounts for the potential viscous flow within the interfacial layer and its stress-dependent behavior. This allows for a more efficient mobilization of hydrocarbons from the organic matrix during pressure depletion. Furthermore, the adsorbed layer moderates the stress sensitivity of organic nanopores, leading to less severe permeability impairment over time compared to the idealized case. Consequently, omitting adsorption effects results in a conservative and likely inaccurate underestimation of well performance, highlighting the necessity of its explicit inclusion in predictive models for shale oil reservoirs.

## 5. Limitations and Future Work

It is important to note that the present model does not account for the viscoelastic behavior of reservoir fluids. The pressure depletion processes can induce time-delayed responses such as creep and relaxation, which are better captured by viscoelastic models (e.g., Maxwell or Jeffreys models). The incorporation of such time-dependent rheology is a complex but valuable extension. Future work will focus on obtaining the necessary experimental data for fluid relaxation times under high-pressure, high-temperature conditions in nanopores and integrating a suitable viscoelastic constitutive equation into the current transport framework to simulate transient production behaviors more accurately.

The current model operates under isothermal and single-phase flow assumptions. While this is a common and practical simplification for analyzing the primary depletion phase in shale reservoirs, where significant temperature gradients within the rock matrix are not typically observed in the short to medium term, it does not account for the known dependencies of viscosity, slippage coefficient, and adsorption layer thickness on temperature and fluid composition. Future model extensions will incorporate thermo-hydro-mechanical (THM) coupling to address thermal effects and multiphase flow for a broader range of recovery processes.

Finally, the current validation lacks direct experimental confirmation of the novel rheological mechanisms (non-Newtonian flow and adsorbed layer mobility). The most compelling validation would involve direct comparison with microfluidic flow experiments in synthetic or real rock chips and rheological measurements of fluids under confinement. While such data is not yet available for the specific systems studied herein, future work will prioritize collaborative efforts to obtain these measurements. The subsequent integration of this experimental data will be essential for calibrating the model parameters and ultimately providing definitive validation of the proposed physics.

Future research will focus on these main extensions of the current work: (1) Incorporating the viscoelastic fluid behavior as noted in Section 2.2.1 and (2) developing a fully coupled THM framework. This framework will explicitly account for temperature-dependent fluid and adsorption properties, as well as stress-induced pore deformation, to more accurately model enhanced oil recovery processes and long-term production behavior in organic-rich shales.

## 6. Conclusions

This study successfully developed and validated a novel microscale FSC numerical framework that explicitly integrates three critical, scale-dependent physical mechanisms: adsorption, slip, and stress sensitivity. This model provides essential quantitative and mechanistic insights into the complex coupled behavior of shale oil reservoirs. The key findings are summarized as follows:

(1) Larger pore diameters and higher porosity promote efficient pressure dissipation, leading to long-term stress relaxation and significantly mitigating long-term permeability decay. This structure-dependent mechanical buffering capacity is crucial for sustained production.
(2) Tortuosity dictates the concentration of stress. Low tortuosity induces severe localized stress concentration along limited flow paths, causing rapid micro-channel closure. Higher tortuosity enforces stress homogenization, which preserves long-term permeability.
(3) The benefit of high fracture permeability is quickly constrained by heterogeneous micro-stress concentration near the fracture faces, resulting in localized mechanical failure and rapid, dominating permeability attenuation in the early stages of depletion.

In essence, this work provides a robust, mechanism-based tool for optimizing unconventional resource development, moving beyond macroscopic assumptions by directly quantifying the impact of nano- and microscale physical processes. Future research will expand this framework to include multi-component transport and thermal effects.

## Figures and Tables

**Table 1:** Summary of model parameters.

Parameter	Value	Parameter	Value
Porosity	0.2	Permeability	100 mD
Initial Reservoir Pressure	42 MPa	Rock Compressibility	1.4 × 10^−3^ MPa^−1^
Oil Density	827.172 kg/m^3^	Oil Saturation	0.8
Water Formation Volume Factor	1.06395	Water Compressibility	5.45 × 10^−7^ MPa^−1^
Water Viscosity	0.232 mPa·s	Water Density	946 kg/m^3^
Young’s Modulus	30 GPa	Poisson’s Ratio	0.3

## Data Availability

The original contributions presented in this study are included in the article. Further inquiries can be directed to the corresponding author.

## Abbreviations

The following abbreviations are used in this manuscript:

FSC	Fluid–Solid Coupling
MINC	Multiple Interacting Continua
BHP	Constant Bottom-hole Pressure
CMG	Computer Modelling Group
THM	Thermo-Hydro-Mechanical

## Nomenclature

Symbol	Description	Unit
Subscript in and or	The inorganic and organic material, respectively	-
*τ*_0_ and *τ*	The yield stress and shear stress, respectively	Pa
*K*	The consistency coefficient	Pa·s^n^
*μ*_b,app_	The shear-dependent apparent viscosity	mPa·s
*β*	The ratio of adsorbed-layer fluid viscosity to boundary-layer fluid viscosity	Dimensionless
*R*	The radius of the inorganic pore	M
*λ*	The slip length	M
*v_b_*	The flow velocity of bulk-phase shale oil	m/s
*R*_e_	The effective flow radius of the pore	M
Δ*p*	The pressure difference across the pore	Pa
*L*	The pore length	M
*C*	The fitting coefficient consolidated	-
*θ*	The contact angle that quantitatively represents the wettability of the fluid–solid system	-
*ρ*	The density of fluid	g/m^3^
*δ*	The thickness of the adsorption layer	M
*δ*_ads_	Total thickness of the adsorbed layer	M
*δ*_v_	Thickness of the viscous interfacial layer	M
*δ*_max_	The maximum adsorbed thickness	M
*h*	Thickness of the immobile adsorbed layer	M
*p_L_*	Langmuir pressure	Pa
*p*	Pore pressure	Pa
γ	Shear rate	1/s
***σ***	The Cauchy stress tensor	Pa
**C**	The fourth-order elastic stiffness tensor	Pa
*ε*	Strain tensor	dimensionless
*α*_B_	Biot coefficient	dimensionless
**I**	The second-order identity tensor	-
**f**	Body force vector	Pa/m
∇	Hamiltonian operator	-
*w_f_* and *w_f0_*	The current and initial hydraulic fracture aperture, respectively	m
*c_f_*	Fracture compressibility coefficient	Pa^−1^
*σ*_n_ and *σ*_n0_	Current and initial effective normal stress acting on the fracture plane, respectively	Pa
*ϕ*_m_ and *ϕ*_m0_	Current and initial matrix porosity	dimensionless
*ε*_v_	Volumetric strain, reflecting the rate of change in rock geometry	dimensionless
*k*_app_	Apparent permeability	D
*k*_m0_	Initial apparent permeability	D
*k*_f_	Current apparent permeability of fracture	D
*k*_f0_	Initial apparent permeability of fracture	D
*c*_p_ and *b*_p_	The empirical stress sensitivity coefficients associated with the shale matrix	dimensionless
